# Neuropharmacology of Nicotine Addiction and Therapeutic Strategies for Smoking Cessation

**DOI:** 10.3390/biology15161412

**Published:** 2026-08-17

**Authors:** Ahmed A. Hefny, Rahul C. Karuturi, Subha Kalyaanamoorthy, Praveen P. N. Rao, Aravindhan Ganesan

**Affiliations:** 1 ArGan’sLab, Department of Chemistry and Biochemistry, Faculty of Science, Wilfrid Laurier University, Waterloo, ON N2L 3C5, Canada; 2 School of Pharmacy, Faculty of Science, Waterloo University, Waterloo, ON N2L 1C5, Canadapraveen.nekkar@uwaterloo.ca (P.P.N.R.); 3 Department of Chemistry, Faculty of Science, Waterloo University, Waterloo, ON N2L 3G1, Canada; subha.kalyaanamoorthy@uwaterloo.ca

**Keywords:** tobacco, nicotine addiction, nicotinic acetylcholine receptors, α4β2 nAChR, smoking cessation, neuropharmacology, varenicline, cytisine, nicotine replacement therapy, receptor-targeted therapies

## Abstract

Tobacco use remains one of the leading preventable causes of morbidity and mortality worldwide, contributing to more than 7 million deaths annually and imposing a substantial economic burden on healthcare systems and global productivity. The addictive properties of tobacco are primarily mediated by nicotine, which exerts its effects through neuronal nicotinic acetylcholine receptors (nAChRs) within brain reward circuits. Among these receptor subtypes, α4β2-containing nAChRs play a central role in nicotine dependence by regulating dopaminergic signaling associated with reinforcement, craving, withdrawal, and relapse. Repeated nicotine exposure induces neuroadaptive changes in receptor expression and neural circuitry, contributing to the chronic and relapsing nature of addiction. Advances in addiction neuroscience and receptor pharmacology have enhanced the understanding of nicotine-mediated signaling and facilitated the development of evidence-based smoking cessation therapies. Current treatment approaches include nicotine replacement therapies, antidepressant-based interventions, and partial nAChR agonists such as varenicline and cytisine. Emerging strategies encompass subtype-selective ligands, allosteric modulators, immunotherapeutics, neuromodulation techniques, and digital health technologies aimed at improving cessation outcomes. This review summarizes the neuropharmacological mechanisms underlying nicotine addiction and critically examines current and emerging therapeutic strategies, highlighting their mechanisms of action, clinical efficacy, limitations, and future potential for tobacco cessation.

## Simple Summary

Tobacco use and nicotine addiction are among the many persisting public health threats. They significantly contribute to premature death, disability, and substantial social and economic burdens worldwide. Although smoking rates have declined in many countries, tobacco use remains prevalent among vulnerable populations, while the increasing use of electronic nicotine products raises concerns, particularly among adolescents and young adults. This review summarizes global patterns and health consequences of tobacco use, explains how nicotine alters brain signaling to promote dependence and repeated tobacco consumption, and evaluates current and emerging approaches for smoking cessation. Nicotine replacement therapies and non-nicotine medications can improve the likelihood of cessation, but there is great variability in individual responses to treatment and relapse is common. Emerging approaches, including combinatorial therapies, nicotine immunizations, non-invasive brain stimulation, mobile applications, artificial intelligence-assisted support and virtual and augmented reality innovations, may help to provide more personalised and accessible methods to help people quit. More research is needed to develop safer, more effective and longer-lasting interventions. Medical intervention coupled with behavioural and technological support could improve smoking cessation outcomes and reduce tobacco health disparities and global impact of nicotine dependence.

## 1. Introduction

Tobacco use remains one of the most prevalent forms of psychoactive substance consumption worldwide, with over one billion individuals regularly using tobacco-related products [1,2,3]. Despite substantial reductions in smoking prevalence across many high-income countries, tobacco consumption continues to represent a major public health challenge because of its strong association with chronic disease, premature mortality, and sustained addiction [4,5]. According to a recent report from the World Health Organization, one-in-five adults worldwide used tobacco in 2024, representing nearly 1.2 billion users globally [1,6]. Tobacco use prevalence varies substantially across regions, with approximately 14% prevalence reported in the Americas compared with nearly 37% in South-East Asia [1,6]. Tobacco has a long history of ceremonial, cultural, and recreational use across different regions; however, the commercialization and mass production of tobacco products have transformed it into a widely available consumer product and contributed substantially to the global burden of tobacco use and nicotine dependence [7,8,9,10,11]. The health consequences of commercial tobacco use are extensive and well established. Tobacco smoke contains a complex mixture of more than 7000 toxicants and around 70 carcinogens, including polycyclic aromatic hydrocarbons, tobacco-specific nitrosamines, carbon monoxide, and particulate matter. Collectively, these constituents contribute to the development of malignancies, cardiovascular disease, chronic respiratory disorders, and widespread systemic dysfunction [12,13,14,15,16].

Nicotine is the principal psychoactive in tobacco which is responsible for the development and maintenance of dependence [17,18,19]. Upon inhalation, nicotine rapidly reaches the brain and activates neural reward pathways, reinforcing repeated use. Nicotine exerts its addictive effects primarily through neuronal nicotinic acetylcholine receptors (nAChRs), a family of ligand-gated ion channels that regulate neurotransmission within brain reward circuits. Among these receptors, α4β2-containing nAChRs play a central role in nicotine reinforcement, dependence, and relapse, making them important therapeutic targets for smoking cessation. Chronic nicotine exposure induces neuroadaptive changes that contribute to tolerance and dependence, while cessation is frequently associated with a well-characterized withdrawal syndrome, including irritability, anxiety, dysphoria, impaired concentration, increased appetite, sleep disturbances, and restlessness [20,21]. These symptoms significantly contribute to the high relapse rates observed among individuals attempting to quit smoking [22,23,24]. Although nicotine is the primary driver of addiction, it is not the main cause of the tobacco-related diseases; this distinction has informed the development of therapeutic strategies aimed at reducing harm while addressing dependence [25,26]. Among these approaches, nicotine replacement therapies (NRTs) have been widely implemented as evidence-based cessation tools that deliver controlled doses of nicotine without exposure to the harmful constituents generated during tobacco combustion [27,28]. Recently, electronic nicotine delivery systems (ENDSs) have emerged as alternative nicotine delivery platforms that may reduce toxicant exposure among individuals who completely transition away from combustible cigarettes [29,30]. Although these products are not risk-free, the lack of tobacco consumption substantially minimizes exposure to many of the toxicants and carcinogens linked to tobacco-related diseases. Several studies suggest that these products have the potential to contribute to tobacco harm reduction, especially in adults who fail to quit smoking via conventional means. In terms of effectiveness, randomized clinical trials and modern Cochrane reviews show that ENDSs containing nicotine have the ability to help people stop smoking cigarettes successfully, even better than traditional NRTs [31]. On the other hand, there is still a question about the effect of such products in the long run on the health of the consumer because of their increasing popularity among teenagers and non-nicotine users, thus raising the fear of addiction to such substances. Therefore, this needs to be balanced against the harm reduction strategy [32,33,34].

This review discusses the neuropharmacological basis of nicotine addiction, with emphasis on the role of nicotinic acetylcholine receptors in reward signaling, dependence, and relapse. Current and emerging therapeutic strategies for tobacco cessation are examined, including nicotine replacement therapies, partial receptor agonists, antidepressant-based therapies, subtype-selective ligands, Immunotherapeutics, and neuromodulation approaches. In addition, digital health technologies, including artificial intelligence-assisted interventions and virtual and augmented reality platforms, have emerged as promising tools for improving tobacco cessation outcomes and are discussed as potential adjuncts to conventional pharmacological and behavioral interventions. Finally, the review highlights current challenges and future directions in the development of more effective nicotinic receptor-targeted therapies for nicotine addiction.

## 2. Epidemiology and Public Health Burden of Tobacco Use

The epidemiology of tobacco use helps identify populations at greatest risk of nicotine dependence, tobacco-related disease, and premature mortality. Patterns of use vary by region, age, socioeconomic status, and health condition, while differences in access to prevention and cessation services further shape outcomes. Understanding these patterns is therefore essential for developing effective public health and treatment strategies.

### 2.1. Global Prevalence, Disease Burden, and Health Disparities

Tobacco use remains one of the most significant preventable causes of disease and death worldwide. Large-scale epidemiological studies, including data from the Global Burden of Disease (GBD) project, estimate that more than 1.1 billion individuals were active smokers in 2019, representing a substantial global health burden despite decades of tobacco control efforts [35,36]. Although age-standardized smoking prevalence has declined in many regions since 1990, population growth and demographic expansion have resulted in a continued increase in the absolute number of tobacco users worldwide [37]. Consequently, tobacco consumption remains deeply entrenched across both developed and developing nations.

Combustible tobacco products continue to account for the majority of tobacco-related morbidity and mortality [37,38]. It has been estimated that tobacco smoking contributes to more than 7 million deaths annually [1,37,39,40,41]. These figures underscore the persistent global impact of tobacco consumption and highlight the limitations of current prevention and cessation strategies in fully addressing nicotine dependence at the population level.

The burden of tobacco use is not evenly distributed. Many high-income countries have achieved substantial declines through taxation, smoke-free policies, public education, advertising restrictions, and improved access to cessation support. By contrast, prevalence remains high in several low- and middle-income countries, where rapid population growth, weaker regulatory systems, and continued tobacco industry marketing sustain consumption [42,43]. As a result, an increasing proportion of tobacco-related disease occurs in regions with limited healthcare resources and less developed tobacco-control infrastructure.

Important inequalities are also observed within countries. Smoking is more common among individuals with lower income, limited education, unstable housing, and restricted access to healthcare [44,45]. Rates are also considerably higher among people living with depression, anxiety, schizophrenia, or substance use disorders [46,47,48]. Altered reward processing, stress sensitivity, and impulsivity may contribute to this association, although social disadvantage and barriers to treatment are equally important [49,50]. These populations often experience greater nicotine dependence and lower cessation success, reinforcing existing health inequalities [51,52,53,54,55,56]. These disparities begin early in life, as most long-term tobacco use is established during adolescence or young adulthood.

### 2.2. Youth Initiation and Vulnerability to Nicotine Dependence

**Figure 1:**
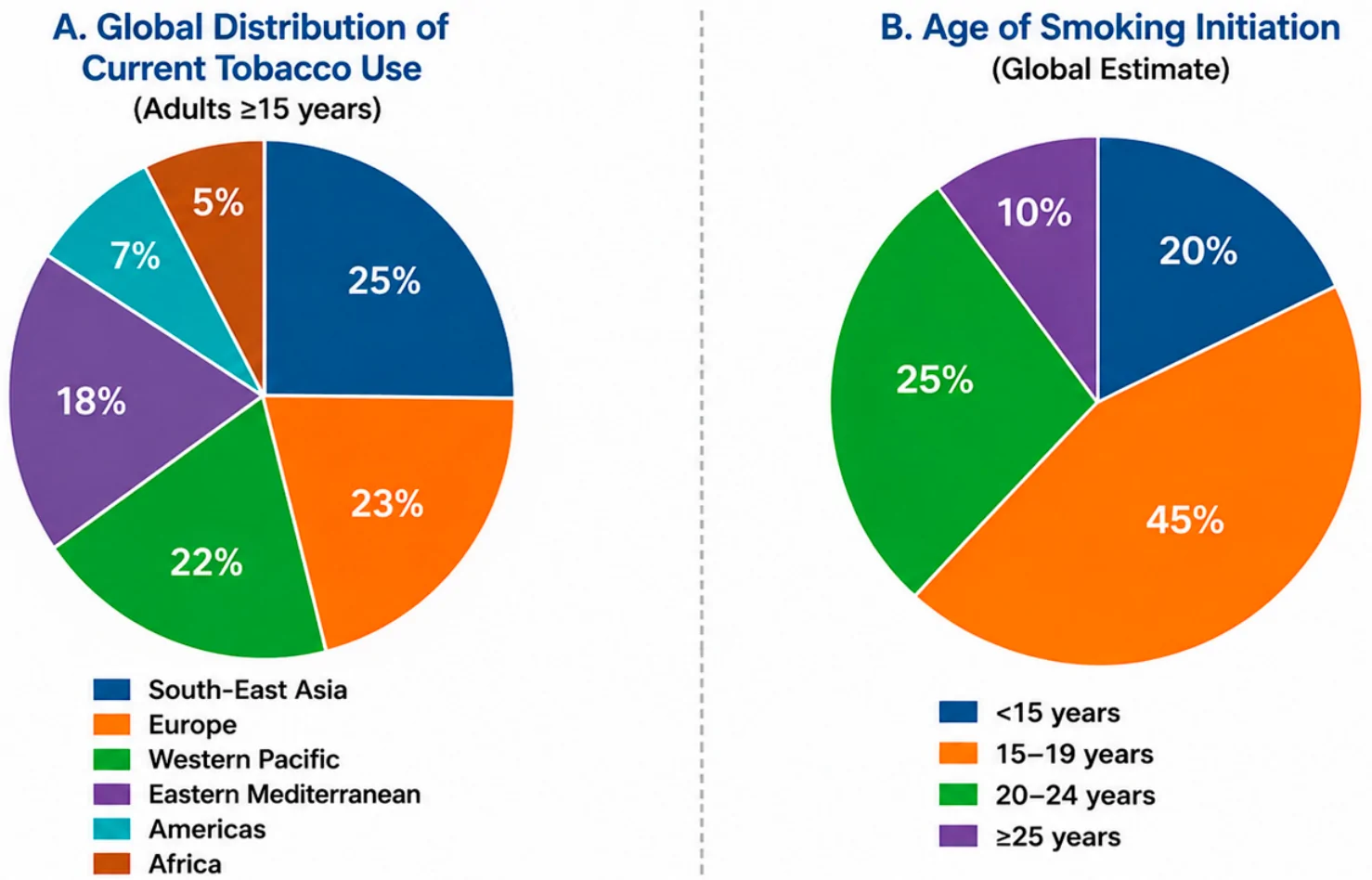
Global burden of tobacco use and smoking initiation patterns. (**A**) Regional distribution of current tobacco use among adults (≥15 years) worldwide. (**B**) Estimated age of smoking initiation, showing that most individuals begin tobacco use before the age of 25 years. The data highlights the persistent global burden of tobacco consumption and the importance of targeting youth and young adults in smoking prevention efforts. Created in Biorender. https://app.biorender.com/illustrations/6a710db436ae82bb4f1f8227?slideId=0d614b07-5d27-4d90-a8f4-32a8f42422c0 (accessed on 23 June 2026).

Tobacco use is most commonly initiated during adolescence and early adulthood, a developmental period characterized by increased risk-taking behaviors and heightened neurobiological vulnerability to addiction. Epidemiological studies consistently demonstrate that the majority of smokers begin tobacco use between 15 and 25 years of age, often establishing patterns of nicotine dependence that persist throughout adulthood, as shown in Figure 1 [57,58]. Adolescence also represents a critical period of brain development during which nicotine exposure may influence the maturation of neural circuits involved in reward processing, cognitive function, and addiction.

Although cigarette smoking among adolescents has declined substantially in many high-income countries, the emergence of ENDSs has altered the landscape of nicotine exposure among youth. The rapid adoption of e-cigarettes and vaping products has become a global public health concern, particularly among adolescents and young adults. Factors such as appealing flavors, targeted marketing, social acceptability, and perceptions of reduced harm have contributed to increased experimentation and regular use [59]. The growing prevalence of ENDS use has raised concerns regarding early nicotine exposure during critical periods of brain development. Nicotine exposure during adolescence may interfere with neural maturation, increase vulnerability to future substance use disorders, and promote long-term dependence. These concerns may be particularly relevant in populations with elevated rates of tobacco use and nicotine dependence [60,61].

In addition to END systems, nicotine pouches have recently emerged as a rapidly expanding category of non-combustible nicotine products. These tobacco-free oral products deliver nicotine through the buccal mucosa and are often marketed in a variety of flavors and nicotine strengths. Their discreet use, perceived reduced harm, and increasing availability have contributed to growing popularity, particularly among adolescents and young adults [62]. Although nicotine pouches eliminate exposure to many toxic combustion-related chemicals found in cigarettes, concerns have been raised regarding their potential to promote nicotine initiation, sustain nicotine dependence, and increase overall nicotine exposure among youth [63]. Consequently, regulatory agencies and public health organizations, including WHO, have emphasized the need for continued surveillance and research to better understand their long-term health effects and public health implications [6].

### 2.3. Health Consequences of Tobacco Use and Benefits of Smoking Cessation

Tobacco use is a major contributor to premature mortality and disability-adjusted life years worldwide. Smoking is strongly associated with lung cancer, chronic obstructive pulmonary disease, ischemic heart disease, stroke, and cancers of the upper aerodigestive tract, bladder, pancreas, and other organs [64,65,66,67]. It is also linked to metabolic dysfunction, impaired immunity, reproductive complications, and greater susceptibility to infectious disease. In regions where smokeless tobacco use is common, particularly parts of South-East Asia, it contributes substantially to oral cancer and oral potentially malignant disorders.

Health risks increase with earlier initiation and longer duration of use. Individuals who begin smoking during adolescence and continue into adulthood experience substantially greater cumulative exposure than those who initiate later or stop earlier [68]. Persistent smokers may lose more than ten years of life expectancy, and up to two-thirds may eventually die from a smoking-related cause [69,70]. These risks can be reduced considerably through cessation. Stopping smoking at any age lowers the likelihood of cardiovascular disease, respiratory illness, cancer, and premature death, although the greatest benefit is achieved when cessation occurs early [71]. Individuals who quit before the age of 40 can avoid most of the excess mortality associated with continued smoking [72,73]. Benefits are also seen among older smokers, including improved respiratory function, better quality of life, and reduced healthcare use [74].

Despite these well-established benefits, cessation remains uneven across populations. Individuals facing socioeconomic disadvantage, psychiatric comorbidity, limited healthcare access, or high nicotine dependence are less likely to achieve long-term abstinence [75,76,77]. Reducing the global burden of tobacco use will therefore require not only broad prevention policies but also accessible pharmacological treatments, behavioral support, and culturally appropriate cessation programs. These persistent differences in treatment response also highlight the need to understand the neurobiological mechanisms that sustain nicotine dependence, which are discussed in the following section.

## 3. Neurobiology of Nicotine Addiction

Nicotine addiction is driven by complex neurobiological mechanisms involving nAChRs, neural reward pathways, and long-term neuroadaptive changes in the brain. The interaction between nicotine and nAChRs alters neurotransmission across multiple neural circuits associated with reinforcement, craving, dependence, and relapse [19,78]. This section discusses the molecular pharmacology of nAChRs and the neural circuitry underlying nicotine addiction.

### 3.1. Molecular Pharmacology of Nicotinic Acetylcholine Receptors

Nicotine is the primary psychoactive constituent of combustible tobacco products, ENDSs, and nicotine pouches [17,79]. Its central and peripheral effects are mediated by nAChRs, a family of pentameric ligand-gated ion channels that are normally activated by acetylcholine. Neuronal nAChRs are assembled from a combination of α2-α7 and β2-β4 subunits, resulting in heteromeric or β2–β4 subunits. Homomeric receptors with different binding affinities, ion conductances, activation kinetics, and desensitization profiles [80,81,82].

**Figure 2:**
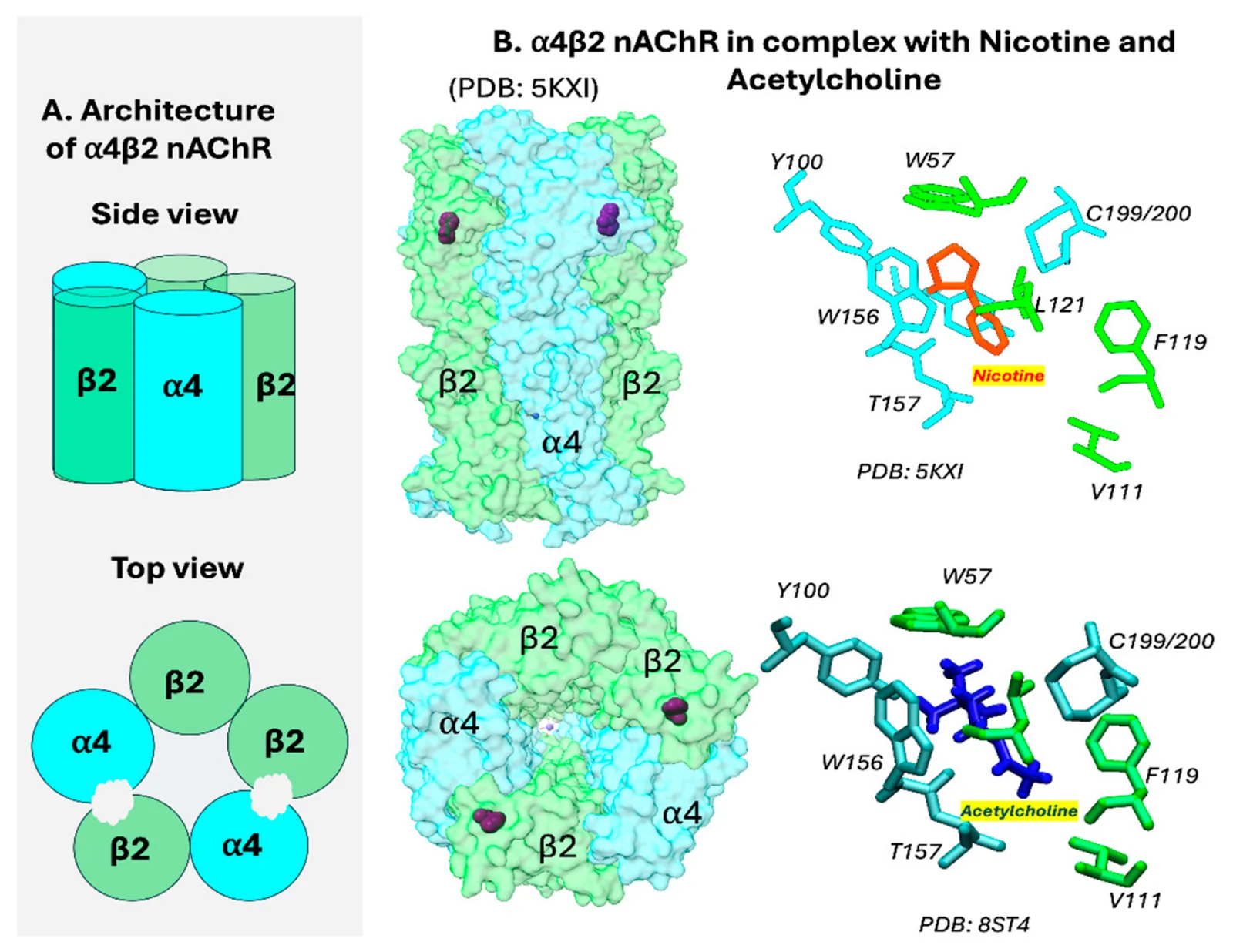
Structural organization and ligand interactions of the α4β2 nAChR. (**A**) Schematic representation of the pentameric architecture of the human α4β2 nAChR shown from side and top views, illustrating the arrangement of α4 and β2 subunits surrounding the central ion-conducting pore. (**B**) Structural models of α4β2 nAChR bound to nicotine (top; PDB: 5KXI) and acetylcholine (bottom; PDB: 8ST4). Surface-rendered receptor structures are shown alongside enlarged views of the ligand-binding pocket at the extracellular α/β subunit interface. Key amino acid residues involved in ligand recognition and stabilization are highlighted. Nicotine, shown in red, and acetylcholine, shown in blue, binding at this extracellular site induces conformational changes that promote channel opening and cation permeation, contributing to nicotine-mediated neurotransmission and addiction.

Among these subtypes, α4β2-containing receptors are highly expressed in the brain and exhibit strong affinity for nicotine, making them especially important in reinforcement and Nicotine dependence [83,84]. In comparison, homomeric α7 receptors have lower nicotine affinity but greater calcium permeability and are involved in synaptic signaling, plasticity, and cognitive function [85]. Structurally, the α4β2 nAChR contains an extracellular ligand-binding domain, a transmembrane ion-conducting pore, and an intracellular region involved in receptor regulation and signaling. Nicotine binds at the extracellular interface between the α and β subunits, in the same orthosteric region recognized by acetylcholine (Figure 2) [86,87].

Ligand binding induces a conformational change that opens the channel and allows the influx of Na^+^ and Ca^2+^. The resulting membrane depolarization can start neuronal firing, while an increase in intracellular Ca^2+^ activates signaling pathways involved in gene regulation and synaptic plasticity [88,89]. nAChRs are located in postsynaptic and presynaptic terminals, where they regulate the release of dopamine, glutamate, γ-aminobutyric acid (GABA), serotonin, and other neurotransmitters [90]. Its wide distribution allows nicotine to affect several interconnected nervous systems instead of acting through a single pathway.

Upon activation, the nAChR enters a desensitized state in which the ligand may remain bound while the channel becomes temporarily unresponsive [91]. The kinetics of desensitization vary between receptor subtypes; β2-containing receptors are basically readily desensitized at nicotine concentrations reached during smoking, while α7 receptors show a different temporal response [91]. With repeated exposure, receptor expression and function change. So chronic nicotine use is mainly associated with upregulation of α4β2-containing receptors in cortical and subcortical areas, possibly through intracellular stabilization and maturation of desensitized receptors [92]. Alterations in β3- and β4-containing receptors have also been reported in the medial habenula–interpeduncular nucleus (MHb-IPN) pathway, helping to mediate nicotine aversion and withdrawal [93,94].

But receptor pharmacology alone does not determine addiction responsibility. The rate at which nicotine reaches the brain is equally important. Cigarette inhalation produces rapid fluctuations in brain nicotine concentrations that strongly reinforce repeated use, whereas slower nicotine administration generally produces weaker reinforcement [95]. These pharmacokinetic differences help explain why combustible cigarettes are more addictive than most therapeutic nicotine formulations [96].

### 3.2. Neural Circuitry, Neuroadaptation, and Withdrawal

The behavioral response to nicotine reflects the integration of rewarding and aversive signals across several brain regions. Nicotine may produce pleasurable effects at lower or familiar doses, while higher doses can induce nausea, dizziness, or dysphoria. The balance between these responses varies according to dose, prior exposure, tolerance, and individual sensitivity [78].

**Figure 3:**
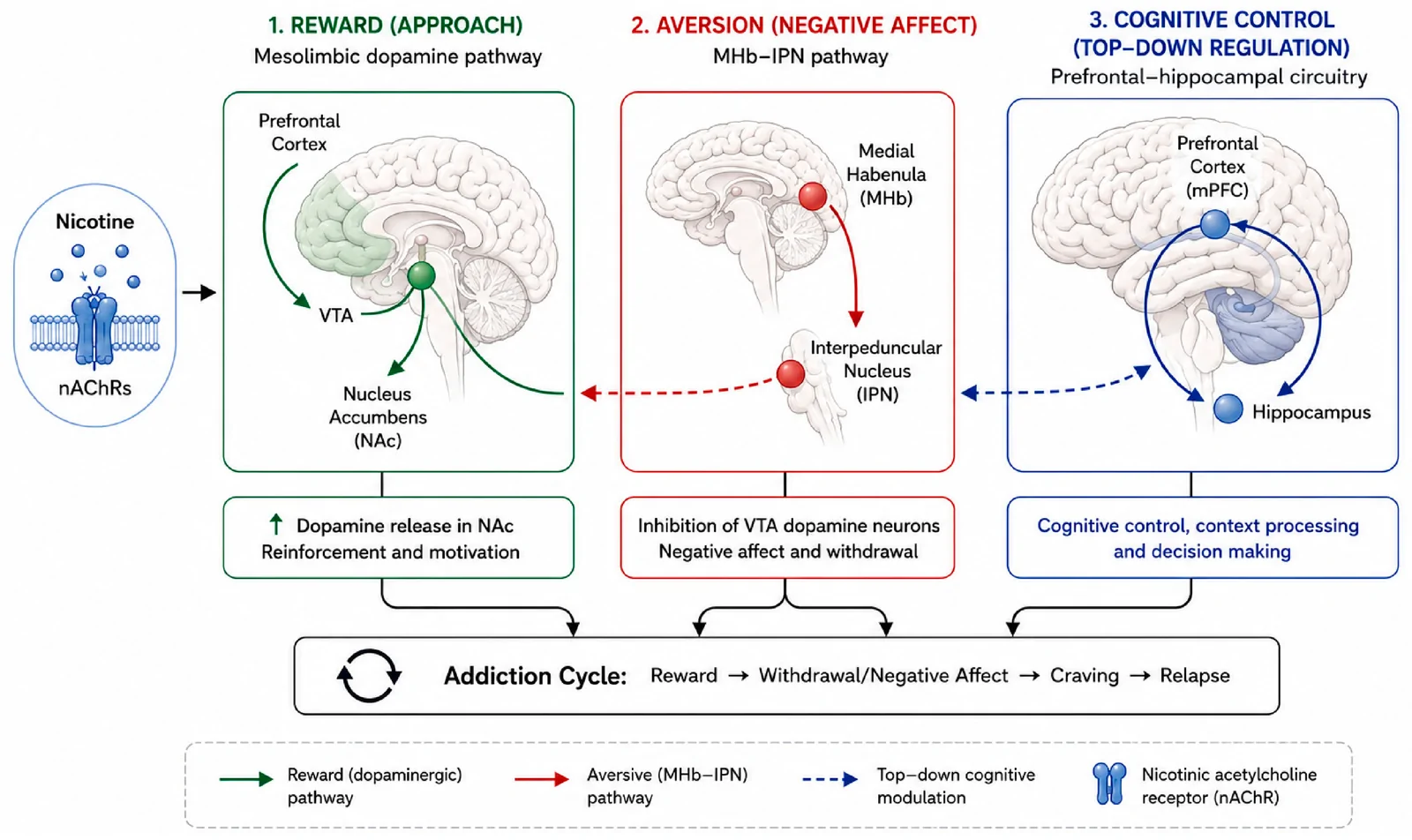
Schematic representation of the neural circuitry underlying nicotine addiction. (1) Reward (approach): Nicotine activates nAChRs within the mesolimbic reward pathway (VTA-NAc), increasing dopamine release and reinforcing nicotine-seeking behaviour. (2) Aversion (negative Affect): The MHb-IPN circuit mediates aversive responses to nicotine and contributes to withdrawal-related negative affect. (3) Cognitive Control (Top-Down Regulation): Prefrontal cortical and hippocampal networks regulate decision-making, contextual memory, craving, and relapse. Together, these circuits drive the addiction cycle of reward, dependence, withdrawal, and relapse. Green arrows represent the mesolimbic reward (dopaminergic) pathway, red arrows indicate the aversive MHb–IPN pathway, and blue dashed arrows denote top-down cognitive modulation. Colored circles identify the principal brain regions involved in each circuit, while the blue receptor icon represents nicotinic acetylcholine receptors (nAChRs). Created in Biorender. https://app.biorender.com/illustrations/6a710e8a01d2800c2af9c77e?slideId=cb680c30-8896-4291-8420-168410f26b36 (accessed on 23 June 2026).

Nicotine reinforcement is largely mediated by the mesolimbic dopaminergic pathway. Dopamine neurons originating in the ventral tegmental area (VTA) project to the nucleus accumbens (NAc) and prefrontal cortex, regions involved in reward, motivation, and decision-making; see Figure 3 [19,97]. Within the VTA, nAChRs are expressed on dopaminergic neurons and on the GABAergic and glutamatergic inputs that regulate them. Nicotine can therefore increase dopamine neuron firing directly while also modifying excitatory and inhibitory control of these cells. The resulting elevation of dopamine in the NAc strengthens the association between nicotine use and reward-related cues [19,98].

Nicotine intake is also constrained by aversion-related pathways. The MHb–IPN circuit is enriched in α5-, β3-, and β4-containing nAChRs and contributes to unpleasant responses at higher nicotine doses [93]. Reward and aversion pathways therefore act together to influence nicotine consumption. Repeated exposure can disturb this balance, strengthening reward-related learning while reducing sensitivity to aversive effects, thereby favoring continued use [84,99].

As nicotine exposure becomes chronic, changes occur in receptor availability, synaptic strength, dopaminergic signaling, and stress-related circuits. These adaptations gradually shift nicotine use from reward-seeking toward dependence and relief of discomfort [84,100]. When nicotine intake is reduced or stopped, dopaminergic tone declines and stress-related signaling increases, producing irritability, anxiety, dysphoria, impaired concentration, sleep disturbance, and craving. This negative affective state is a major contributor to relapse, as nicotine is often resumed to relieve withdrawal rather than to reproduce the initial pleasurable response [101]. Together, these findings show that nicotine addiction develops through the combined effects of receptor activation, rapid drug delivery, reward learning, aversion processing, and long-term neural adaptation. This mechanistic complexity also helps explain why treatments directed at a single receptor subtype or pathway may reduce some features of dependence without fully preventing relapse.

## 4. Pharmacological Management of Nicotine Addiction

**Figure 4:**
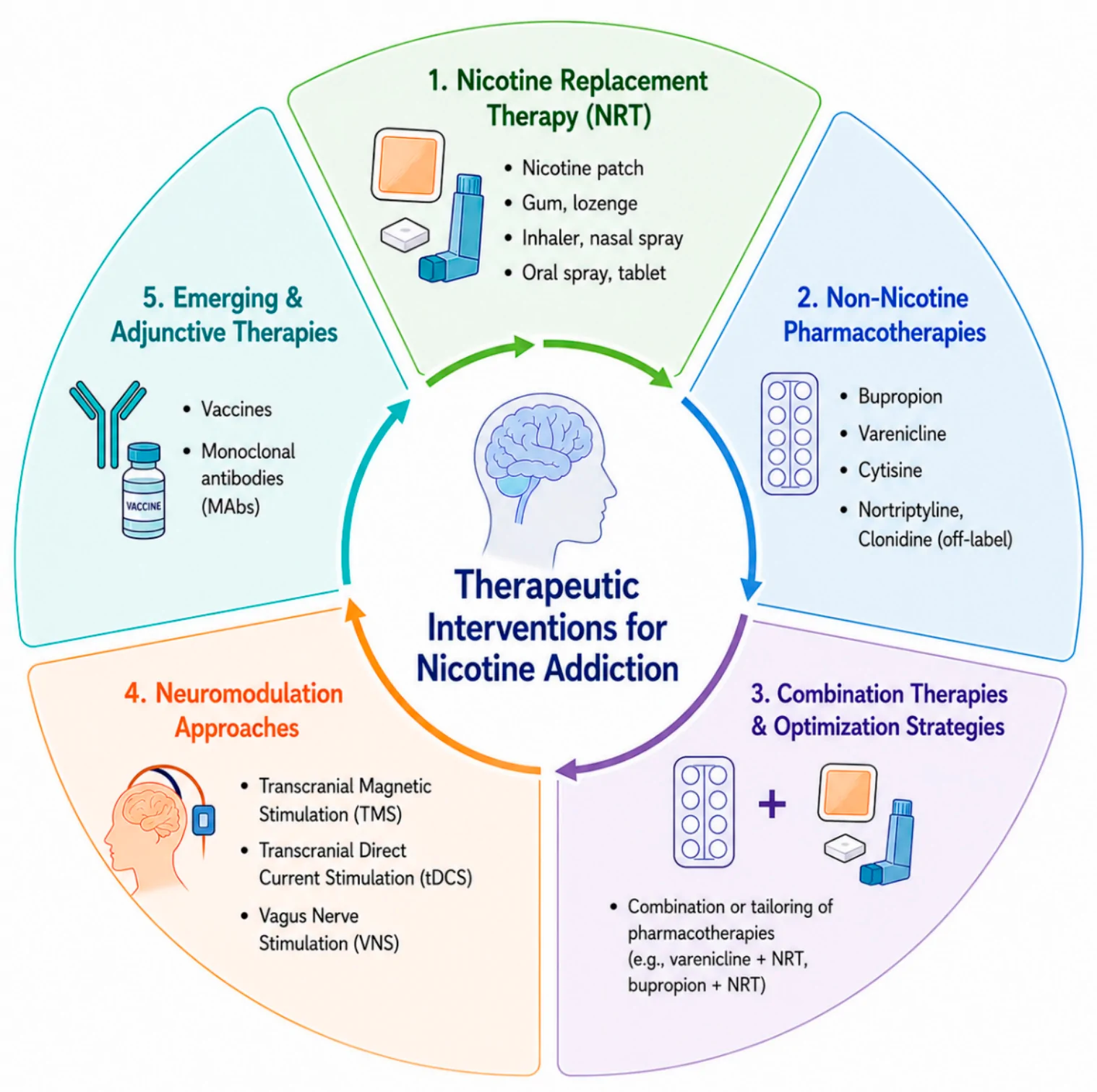
Comprehensive overview of current and emerging therapeutic interventions for nicotine addiction. The major treatment strategies for tobacco cessation are categorized into: (1) NRT, including transdermal patches, chewing gum, lozenges, inhalers, nasal sprays, and oral formulations (2) non-nicotine pharmacotherapies, including varenicline, cytisine, bupropion, and related agents targeting nicotinic receptor signaling and reward pathways; (3) combination and optimization strategies that integrate multiple pharmacological approaches to enhance cessation efficacy; (4) neuromodulation techniques, including TMS, tDCS, and VNS, which modulate addiction-related neural circuits; and (5) emerging adjunctive therapies, such as nicotine vaccines and monoclonal antibodies, aimed at reducing nicotine reinforcement and dependence. Created in Biorender. https://app.biorender.com/illustrations/6a710f40db81557f8260fa64?slideId=9e5578e2-282c-46b2-ab0d-6bfd73504bdf (accessed on 23 June 2026).

Besides behavioural support and counselling, the development of effective pharmacological interventions remains an important strategy for tobacco cessation. Current market-approved therapies primarily focus on reducing nicotine craving, alleviating withdrawal symptoms, and attenuating the reinforcing effects of nicotine by targeting nAChRs and associated neurotransmitter systems. Over the past few decades, advances in the understanding of nicotine addiction and neuropharmacology have contributed to the development of multiple therapeutic approaches, including NRTs, partial nicotinic receptor agonists such as cytisine and varenicline, and antidepressant medications including atypical antidepressants such as bupropion and tricyclic antidepressants such as nortriptyline, as shown in Figure 4. More recently, emerging approaches including subtype-selective ligands, positive allosteric modulators, Immunotherapeutics, and neuromodulation strategies have gained increasing attention as potential next-generation interventions for nicotine dependence. This section discusses established pharmacotherapies and emerging therapeutic strategies for tobacco cessation, focusing on their pharmacological mechanisms, therapeutic efficacy, and clinical relevance.

### 4.1. Nicotine Replacement Therapy

NRT encompasses a range of formulations designed to deliver nicotine in controlled, non-combustible forms. These include transdermal patches, chewing gums, lozenges, mini-lozenges, nasal sprays, and inhalers. The primary mechanism of NRT is to attenuate withdrawal symptoms and cravings by maintaining moderate nicotine levels without producing the rapid pharmacokinetic spikes associated with cigarette smoking [102,103]. Clinical trials have consistently demonstrated that NRT can significantly increase cessation success, typically doubling the likelihood of long-term abstinence relative to no treatment [104,105]. The major NRT formulations, their mechanisms of action, dosing strategies, and commonly reported adverse effects are summarized in Table 1.

#### 4.1.1. Transdermal Patch

Nicotine transdermal patches deliver nicotine through the skin at a slow, sustained rate, resulting in relatively stable plasma concentrations compared with acute nicotine replacement formulations [106]. Available in multiple dosages and wearing durations (e.g., 16- and 24-h systems), patches enable individualized treatment based on dependence level and support gradual dose reduction over several weeks, facilitating physiological adaptation to decreasing nicotine intake [107]. Studies suggest that while stepwise tapering provides symptom relief during nicotine withdrawal, variations in discontinuation strategy (abrupt versus gradual) do not significantly influence cessation outcomes [108]. A key advantage of patch therapy is its simplicity of use, which enhances adherence relative to more actively administered nicotine replacement products. Long-term use of nicotine patches has been shown to be safe for tobacco cessation purposes, with the most commonly reported adverse effects including local skin reactions and sleep disturbances. Although effective in reducing baseline craving and withdrawal symptoms, nicotine patches appear less effective in attenuating cue-induced craving, which may contribute to relapse risk and supports the use of combination therapy with short-acting nicotine replacement formulations [108].

#### 4.1.2. Short-Acting Nicotine Products

Acute dosing nicotine products include nicotine gum, lozenges, sublingual tablets, oral inhalers, and nasal sprays (Table 1). Unlike transdermal patches, these formulations allow users to self-administer nicotine on a flexible, as-needed basis, with both dose and timing titrated according to individual craving intensity and nicotine dependence [103]. This enables their use as “rescue medication” during acute craving episodes, which are strongly associated with relapse risk [109,110]. However, clinical effectiveness depends on adequate and frequent dosing, and underuse is common in real-world settings. Consequently, suboptimal adherence and early discontinuation may limit the therapeutic benefit of acute NRTs, while higher and more frequent dosing has been associated with improved cessation outcomes [111].

#### 4.1.3. Gum

Nicotine polacrilex, commonly known as nicotine gum, was the first form of NRT introduced for consumer use as a transmucosal delivery system [106]. It delivers nicotine via the oral mucosa and is used by intermittent chewing followed by holding the gum in the mouth to facilitate nicotine release. It is available in 2 mg and 4 mg strengths, with higher doses shown to be more effective in more dependent smokers. Use is gradually reduced over time until discontinuation, and absorption of nicotine may be interfered with by concurrent intake of acidic beverages [107,108].

#### 4.1.4. Lozenge

Nicotine lozenges are available in 2 mg and 4 mg formulations and dissolve slowly in the mouth over approximately 30 min. Similar to nicotine gum, they deliver nicotine via the buccal mucosa into the systemic circulation but do not require chewing, making them a suitable alternative for individuals who prefer not to use gum. They allow intermittent, controllable dosing and may provide slightly higher nicotine exposure per dose compared to gum [108,112].

#### 4.1.5. Sublingual Tablet

Nicotine sublingual tablets are designed to be placed under the tongue, where nicotine is absorbed sublingually. Typically available in 2 mg strength, they provide nicotine exposure comparable to that of nicotine gum or lozenges while requiring less active user involvement, as no chewing is needed. Use is recommended for at least 12 weeks, followed by gradual tapering [107,113].

#### 4.1.6. Oral Inhaler

The nicotine inhaler consists of a mouthpiece and a plastic cartridge containing nicotine and is designed to mimic the hand-to-mouth behavioural aspects of smoking while alleviating withdrawal symptoms. Despite its name, most nicotine is delivered to the oral cavity rather than the lungs, with absorption occurring primarily through the oral mucosa at a rate similar to that of nicotine gum [108,114].

#### 4.1.7. Nasal Spray

Nicotine nasal spray is a rapid-acting form of NRT designed to deliver nicotine more quickly than other formulations. It is administered via a multidose pump bottle, with each dose consisting of two squirts, one into each nostril, delivering approximately 1 mg of nicotine per dose [106,107].

**Table 1:** Overview of NRT formulations used in tobacco cessation. The table summarizes currently available NRT products, including transdermal patches, chewing gum, lozenges, sublingual tablets, inhalers, and nasal sprays, together with their mechanisms of nicotine delivery, available strengths, recommended dosing regimens, and commonly reported adverse effects [115].

Delivery Method	Type/Strength	Mechanism	Standard Dosage & Taper	Common Side Effects
Patch	Long-acting 7, 14, 21 mg/d	Provides continuous nicotine absorption through the skin, producing steadier nicotine levels and reducing withdrawal symptoms and cravings.	Start with 21 mg/day for heavier smokers (>10 cigarettes/day), then step down to lower strengths over 8–12 weeks until discontinued.	Skin irritation, insomnia
Gum	Short-acting 2 mg or 4 mg	Intermittently chewed and held in the mouth for about 30 min to release nicotine for absorption through the oral mucosa.	4 mg is recommended if the first cigarette is within ≤30 min of waking; then gradually taper use over 8–12 weeks until discontinued.	Mouth irritation, hiccups, and dyspepsia
Lozenge	Short-acting 2 mg or 4 mg	Dissolves in the mouth and delivers nicotine via buccal mucosal absorption into systemic circulation.	4 mg for the first cigarette ≤30 min after waking, then taper over 8–12 weeks until cessation.	Nausea or Heartburn
Sublingual tablet	Short-acting 2 mg	Held under the tongue, where nicotine is absorbed sublingually into systemic circulation without chewing.	Use for a minimum of 12 weeks, followed by gradual tapering.	Mouth soreness
Inhaler	Short-acting 10 mg nicotine per cartridge	Delivers nicotine via puffing from a cartridge, with absorption mainly through the oral mucosa.	Used as needed; usually ≥6 cartridges/day initially (up to 16/day max), then gradually reduced over time until cessation.	Local irritation of the mouth and throat
Spray	Short-acting 0.5 mg/spray	Rapid delivery of nicotine via nasal spray with absorption through the nasal mucosa into systemic circulation.	1–2 doses/hour as needed, minimum 8 doses/day for first 6 weeks (max 40/day), then gradual taper to cessation.	Nasal irritation

### 4.2. Partial Nicotinic Receptor Agonists

Partial nicotinic receptor agonists represent an important family of non-nicotine drugs that aid in tobacco cessation. Unlike NRTs, these small-molecule agonists directly target nAChRs, particularly the α4β2-containing receptor subtypes. Their targeted binding partially stimulates the nicotinic receptor activity while simultaneously limiting nicotine binding and dopaminergic reinforcement. This dual mechanism helps reduce nicotine craving and withdrawal symptoms while attenuating the rewarding effects associated with cigarette smoking [116]. Among currently available agents, cytisine and varenicline are the most extensively studied partial agonists for nicotine dependence treatment.

#### 4.2.1. Cytisine

Cytisine (also known as cytisinicline) is a natural plant alkaloid extracted from *Cytisus laburnum* that acts as a partial agonist at α4β2 nAChR. It prevents the binding of nicotine to these receptors, thereby reducing nicotine reward and alleviating withdrawal symptoms [114,117]. Cytisine and varenicline share a similar mechanism of action; however, cytisine exhibits lower binding affinity for α4β2 receptors relative to varenicline and has limited blood–brain barrier penetration, resulting in lower central nervous system (CNS) concentrations compared with nicotine and varenicline. It also exhibits poor gastrointestinal permeability, contributing to low bioavailability, and has a short elimination half-life [118,119]. Rigotti et al. reported that cytisine significantly increased tobacco cessation rates compared with placebo, with approximately 3–4-fold higher odds of abstinence at the end of treatment and sustained benefits through 24 weeks, accompanied by reductions in nicotine craving and a favourable safety profile with no treatment-related serious adverse events [120]. In a randomized non-inferiority trial, Courtney et al. found that cytisine failed to demonstrate non-inferiority to varenicline for tobacco cessation, although differences in abstinence rates were small and cytisine was associated with fewer adverse events [121].

More recently, cytisine has been evaluated in large, multisite, phase 3 randomized clinical trials as part of the Ongoing Research of cytisine for Addiction (ORCA) program. In the ORCA-2 trial, Rigotti et al. demonstrated that both 6- and 12-week cytisine regimens, combined with behavioural support, significantly improved biochemically verified smoking abstinence compared with placebo, with benefits sustained through 24 weeks and a favourable tolerability profile [122]. These findings were subsequently confirmed in the ORCA-3 phase 3 replication trial, which demonstrated significant improvements in abstinence outcomes, reductions in nicotine craving, and no treatment-related serious adverse events [120]. Collectively, these phase 3 trials demonstrate the efficacy and tolerability of cytisine for smoking cessation and support its potential as an emerging pharmacological option for tobacco dependence.

#### 4.2.2. Varenicline

Varenicline is a selective partial agonist of nAChRs, derived from cytisine, with high affinity for the α4β2 receptor subtype [123]. It exhibits an equilibrium binding affinity of approximately 0.15 nM in the human cortex and prevents nicotine from binding at α4β2 nAChRs, thereby functionally antagonizing nicotine-induced dopaminergic activation [123,124]. Varenicline demonstrates marked selectivity for α4β2 receptors, with approximately 500–20,000-fold greater affinity than for other nAChR subtypes [125]. As a partial agonist, varenicline elicits lower dopaminergic activation than nicotine, reflecting reduced intrinsic efficacy at α4β2 receptors. Preclinical studies indicate that varenicline reduces nicotine self-administration by up to 50%, suggesting attenuation of nicotine reinforcement [126]. McColl et al. demonstrated that human abuse liability studies show low abuse potential for varenicline, with no significant differences from placebo in drug-liking and drug-high ratings among smokers, and only isolated non-reinforcing subjective effects in non-smokers that were offset by adverse effects [127]. Guo et al. conducted a network meta-analysis of randomized controlled trials (RCTs) and reported that varenicline monotherapy was superior to bupropion, NRT, counselling, and placebo in achieving tobacco cessation, with combination therapy with bupropion potentially providing additional benefit compared with varenicline monotherapy [128].

#### 4.2.3. Dianicline

Dianicline (SSR591813) is a selective α4β2 nAChR partial agonist developed for tobacco cessation. Rollema et al. demonstrated that dianicline binds selectively to α4β2 receptors but exhibits relatively low functional efficacy (~8% of the maximal acetylcholine response) compared with varenicline (22%) [129]. Moreover, dianicline required substantially higher concentrations (10–100 µM) to achieve receptor activation and desensitization compared with varenicline and cytisine (0.01–10 µM range), indicating reduced functional potency. Pharmacokinetic modelling predicted unbound human brain concentrations of approximately 37–84 nM following therapeutic dosing, which falls at the lower end of the range required for significant α4β2 receptor modulation. Consistent with this, dianicline was more than 100-fold less potent than varenicline in stimulating mesolimbic dopamine turnover in the nucleus accumbens, indicating weak modulation of nicotine reward pathways. In a randomized placebo-controlled tobacco cessation trial, dianicline was well tolerated but failed to demonstrate efficacy in achieving sustained abstinence [130]. Although reductions in craving and nicotine withdrawal symptoms were observed early in treatment, these effects did not translate into improved abstinence during follow-up. Consequently, dianicline was discontinued from further clinical development.

#### 4.2.4. ABT-089

ABT-089 is a partial agonist at α4β2 nAChR investigated for its effects on nicotine-seeking behaviour. Lee et al. demonstrated that acute administration of ABT-089 (0.12–12 mg/kg) did not alter nicotine self-administration in rats [131,132]. In contrast, ABT-089 dose-dependently attenuates nicotine- and cue-induced reinstatement of nicotine-seeking behaviour following a systemic nicotine priming injection. Significant reductions in nicotine reinstatement are observed at the highest dose tested (12 mg/kg). These findings indicate that acute ABT-089 reduces reinstatement of nicotine-seeking behaviour without altering nicotine self-administration under the conditions tested [131]. ABT-089 has been evaluated in human clinical studies and was generally well tolerated; however, its efficacy as a tobacco cessation pharmacotherapy has not been demonstrated in clinical trials [133].

#### 4.2.5. TC-2559

TC-2559 is a selective partial agonist of neuronal nAChR of the α4β2 subtype that has been characterized in both in vitro and in vivo studies. In vitro, TC-2559 competes with [^3^H]-nicotine binding in rat brain membrane preparations with high affinity (Ki ≈ 5 nM) and demonstrates functional activity at central nicotinic receptors, including stimulation of dopamine release from rat striatal synaptosomes [134]. In vivo electrophysiological recordings in anesthetized rats show that systemic administration of TC-2559 increases both the firing rate and bursting activity of dopaminergic neurons in the ventral tegmental area. These effects are approximately equivalent in magnitude to those produced by nicotine [135].

### 4.3. Antidepressant-Based Therapies

Apart from NRTs and partial nicotinic receptor agonists, several antidepressant medications have also been shown to help with tobacco cessation. However, unlike the partial agonist class that directly act on the nicotinic receptors, antidepressants mainly influence monoaminergic neurotransmission, particularly dopaminergic and noradrenergic signalling pathways involved in reward, mood regulation, and withdrawal responses [136]. These agents may reduce nicotine craving, alleviate withdrawal-associated negative affect, and attenuate relapse risk during tobacco cessation. Among the antidepressants investigated for nicotine dependence treatment, bupropion and nortriptyline are the most extensively studied and clinically utilized agents.

#### 4.3.1. Bupropion

Bupropion, originally developed as an antidepressant, exerts its therapeutic effects in tobacco cessation through inhibition of dopamine and norepinephrine reuptake, as well as non-competitive antagonism of nAChRs. This dual mechanism contributes to reduced craving and attenuation of withdrawal-related mood disturbances. Preclinical studies indicate that bupropion exhibits relatively greater potency at dopamine transporters compared to noradrenaline transporters. Furthermore, chronic bupropion administration has been shown to enhance subsequent dopaminergic responses in the nucleus accumbens upon re-challenge, suggesting adaptive changes in mesolimbic reward circuitry [137,138]. In addition, bupropion exerts weaker effects on noradrenergic systems through inhibition of norepinephrine reuptake. Evidence suggests modulation of neuronal activity within the locus coeruleus, potentially involving α2-adrenergic autoreceptor-mediated regulation. This may contribute to attenuation of stress and negative affect during nicotine withdrawal, thereby supporting tobacco cessation through reduction in withdrawal-related symptoms [137,139,140]. Beyond monoaminergic effects, bupropion also acts directly on nicotinic receptors. Studies demonstrate that it functions as a non-competitive antagonist of nAChRs, with functional relevance primarily at α4β2-containing subtypes, thereby reducing nicotine-evoked receptor activation and downstream neurotransmitter release. This inhibition of nicotinic signalling provides an additional mechanism by which Bupropion may reduce nicotine reinforcement and support tobacco cessation [141]. The IC_50_ for inhibition of nicotinic receptor-mediated currents in rat VTA neurons (~0.28 μM) is lower than peak plasma concentrations observed in humans (~0.5–1 μM), suggesting that clinically relevant exposure is sufficient to engage this mechanism [142]. Meta-analyses of RCTs consistently report odds ratios in the range of approximately 2.0–2.7 for sustained-release bupropion (bupropion SR, 300 mg/day), indicating a two-fold increase in tobacco cessation rates compared with placebo, although results vary across study designs and follow-up periods [143].

#### 4.3.2. Nortriptyline

Nortriptyline is a tricyclic antidepressant, although its efficacy in tobacco cessation does not appear to be related to its antidepressant actions. It is thought to act primarily through noradrenergic modulation, potentially substituting for nicotine-induced noradrenergic activity and thereby attenuating withdrawal-related symptoms [144]. A weak and non-primary role of nAChR antagonism has also been proposed, although evidence for direct receptor-level effects remains limited. Preclinical evidence from rodent models indicates that nortriptyline attenuates nicotine withdrawal-related somatic signs, potentially via modulation of noradrenergic pathways; however, these effects were observed at doses associated with reduced locomotor activity, suggesting a possible non-specific effect [145]. Wagena et al. demonstrated that nortriptyline approximately doubles prolonged abstinence rates compared with placebo and shows efficacy broadly comparable to bupropion sustained release [146]. Although generally reported as well tolerated in clinical trials, its use in routine practice remains limited by concerns regarding adverse effects and lack of first-line regulatory approval. Consequently, nortriptyline is recommended in most clinical guidelines as a second-line pharmacotherapy for tobacco cessation, reserved for individuals who fail to respond to or cannot tolerate first-line agents such as NRT or bupropion. Collectively, these pharmacotherapies have been shown to approximately double to triple cessation success rates compared to placebo, underscoring their importance in clinical practice. The reported α4β2 nAChR binding and inhibition parameters for representative partial nicotinic receptor agonists and antidepressant-based therapies are summarized in Table 2.

### 4.4. Combination Therapies and Optimization Strategies

Evidence from clinical trials indicates that combining pharmacological approaches can further enhance cessation outcomes. For example, the concurrent use of a long-acting nicotine patch with a short-acting NRT formulation (such as gum or lozenges) provides both baseline nicotine levels and rapid relief of breakthrough cravings, resulting in improved abstinence rates compared with monotherapy. A Cochrane systematic review by Theodoulou et al. indicates that combination NRT increases quit rates compared with single-form therapy, and that this finding is consistent across different types of single-form NRT [151]. The review further notes that, although there is high-certainty evidence for combination NRT compared with single-form therapy, the evidence base across other comparisons is less certain, particularly for different dosing strategies, duration of use, and timing of administration. Further research is therefore needed to strengthen and refine findings in these areas. A narrative review by Sweeney et al. similarly supports the rationale for combination NRT, highlighting that while it is associated with improved cessation outcomes, important uncertainties remain regarding optimal dosing strategies, timing of use, tapering protocols, and appropriate product combinations, which limit precise clinical standardization [152]. Beyond demonstrating greater efficacy compared with single-form NRT, combination NRT has also demonstrated efficacy comparable to varenicline. In a randomized clinical trial directly comparing nicotine patches, varenicline, and combination NRT, Baker et al. found no significant differences in biochemically verified abstinence rates between treatment groups at 26 or 52 weeks. These findings suggest that combination NRT represents an effective first-line pharmacological option with comparable long-term cessation outcomes to varenicline [153].

A systematic review and meta-analysis by Wang et al. found that combining NRT with bupropion significantly improved short-term tobacco cessation compared with bupropion monotherapy [154]. However, this benefit was not sustained at long-term follow-up (≥6 months), where no significant difference was observed between groups. Safety profiles were generally comparable, although a higher incidence of nausea was reported with combination therapy. These findings suggest a potential short-term advantage of combination pharmacotherapy, with uncertain long-term superiority. In contrast, a pragmatic RCT conducted by Stapleton et al. in the UK National Health Service tobacco cessation clinics found no evidence of a difference in 6-month abstinence rates between bupropion, NRT, or their combination when delivered alongside behavioural support [155]. The study further suggested a possible subgroup effect favouring bupropion in individuals with a history of depression; however, overall combination therapy did not demonstrate additional benefit over monotherapy in this real-world effectiveness setting.

An RCT by Koegelenberg et al. found that combining varenicline with NRT improved abstinence rates at 12 and 24 weeks and at 6-month follow-up in some outcomes compared with varenicline monotherapy [156]. However, the mechanisms underlying any potential additive effect remain unclear, and findings across studies are inconsistent. A large RCT by Baker et al. found no significant differences in abstinence at 52 weeks between varenicline plus nicotine patch and varenicline alone, and no benefit from extending varenicline treatment duration from 12 to 24 weeks [157]. Overall, these findings suggest that neither combination therapy nor extended varenicline treatment provides a clear additional benefit over standard monotherapy. An RCT by Ebbert et al. reported some improvement in prolonged abstinence at 12 and 26 weeks with combination therapy, but no significant differences in 7-day abstinence or at 52 weeks, and higher rates of anxiety and depressive symptoms [158]. In contrast, an RCT by Cinciripini et al. found no overall improvement in abstinence rates with combination therapy compared with varenicline alone and concluded that the evidence does not support its use as a first-line treatment, despite limited subgroup effects in highly dependent smokers [159]. Overall, evidence from RCTs suggests that combination pharmacotherapy for tobacco cessation shows inconsistent benefits, with some short-term improvements but no consistent long-term superiority over varenicline monotherapy.

### 4.5. Next-Generation nAChR Ligands

Early work by Peter Imming et al. (2001) demonstrated that relatively minor structural modifications of the cytisine scaffold can substantially alter its interaction with nAChRs [160]. Small substitutions produced marked changes in binding affinity and receptor selectivity; for instance, 3-bromocytisine exhibited exceptionally high affinity (*K_i_* = 10 pM), approximately tenfold greater than cytisine (*K_i_* = 0.122 nM). Similarly, replacement of the lactam oxygen with sulphur (thiocytisine) preserved strong binding to α4β2 receptors (*K_i_* = 0.832 nM) while dramatically reducing affinity for α7 receptors (*K_i_* = 4000 nM), corresponding to an approximately 4800-fold subtype preference. These findings indicate that relatively small structural changes can produce large differences in both binding affinity and receptor subtype selectivity.

**Figure 5:**
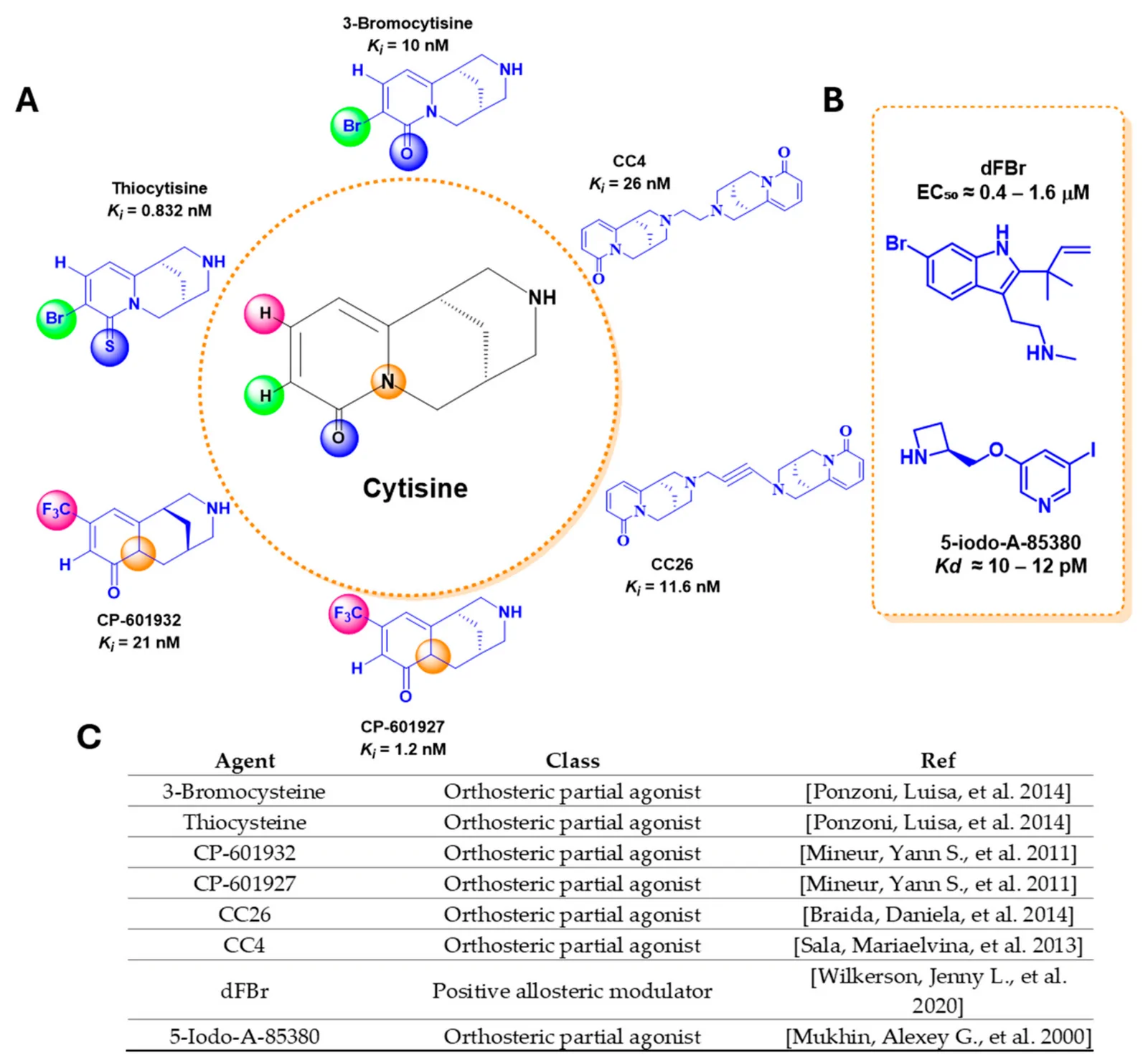
Representative orthosteric and allosteric ligands targeting α4β2 nicotinic acetylcholine receptors. (**A**) Cytisine-derived analogues developed through structure-activity relationship (SAR) optimization for tobacco cessation therapy. Colored circles identify the principal structural modification sites explored during lead optimization, including halogen substitution (green), carbonyl/thiocarbonyl bioisosteric modifications (blue), heterocyclic ring or linker modification sites (orange), hydrogen substitution (magenta), and trifluoromethyl (CF_3_) substitution (pink). (**B**) Positive allosteric modulators (PAMs) of α4β2 nAChRs showing reported binding affinities and functional activities. (**C**) The inset table summarizes the pharmacological classification of each compound together with the corresponding literature references. Pharmacological values were obtained from the cited studies and may reflect different experimental endpoints and assay methodologies [161,162,163,165,168,172].

Building on these structure-activity relationships (SARs), subsequent studies have examined how such modifications translate into functional and behavioural effects. Cytisine-derived ligands illustrate how structural modification can alter nicotinic receptor pharmacology. Luisa Ponzoni et al. (2014) demonstrated that CC4 and the derivative CC26 reduce nicotine-induced conditioned place preference (CPP) in zebrafish, as depicted in Figure 5 [161,162]. Binding studies indicated that these compounds act competitively with nicotine at nAChRs, and when co-administered, they abolished or reversed nicotine’s reinforcing effects, as well as its slight aversive effects, depending on dose [163]. Quantitatively, nicotine produced maximal reinforcement at 0.001 mg/kg, whereas CC26 and CC4 required approximately 10-fold (0.01 mg/kg) and 100-fold (0.1 mg/kg) higher doses, respectively; in contrast, cytisine and varenicline required 1000- to 2500-fold higher doses [133]. Electrophysiological assays in oocyte-expressed receptors showed that CC4 and CC26 inhibit acetylcholine-induced currents at heteromeric nAChRs, likely involving α4β2-containing receptors, with little effect on α7 receptors. Notably, these compounds induced CPP when administered alone but reduced nicotine-induced CPP when co-administered. Therefore, these findings indicate that nicotinic receptor subtype selectivity and competitive ligand interactions are involved in the modulation of nicotine-induced reward in this model.

Although preclinical findings for cytisine-derived ligands have been promising, their translation into clinically effective tobacco cessation therapies has been limited [161]. Within Pfizer’s nAChR drug development programme, several cytisine-derived compounds, including CP-601927 and CP-601932, were advanced as α4β2-targeting partial agonists [164,165]. CP-601932 exhibits high affinity at α4β2 nAChRs but very low efficacy (~2%), resulting in functionally antagonistic activity at this receptor subtype. CP-601932 and CP-601927 were reported as safe in human clinical studies; however, CP-601932 was discontinued following Phase II evaluation due to a lack of efficacy compared with varenicline [166].

Positive allosteric modulation (PAM) of α4β2 nAChRs has emerged as a novel strategy for reducing nicotine intake. In a rat model of nicotine self-administration, the α4β2 PAM desformylflustrabromine (dFBr) reduced nicotine self-administration by approximately 48.3% at 6 mg/kg, without affecting food-reinforced responding [167,168]. Pharmacokinetic analysis showed that approximately one-third of systemically injected dFBr crossed the blood–brain barrier (BBB), with an estimated elimination half-life of 8.6 h [169]. Importantly, dFBr did not substitute for nicotine in self-administration tests, indicating a lack of reinforcing properties. In contrast, the α4β2-selective agonist 5-iodo-A-85380 also reduced nicotine self-administration and exhibited approximately 150-fold selectivity for α4β2 receptors over nicotine [168]. However, orthosteric agonist-based approaches are limited by issues such as receptor adaptation and compensatory behavioural responses, which have driven interest in alternative strategies such as PAM [170,171]. Further studies using drug discrimination paradigms may help determine whether dFBr produces nicotine-like subjective or interoceptive effects.

### 4.6. Emerging and Technology-Assisted Therapeutic Strategies

Despite advances in conventional pharmacotherapies, combination treatment strategies, and nAChR-targeted drug development, long-term smoking abstinence remains difficult for many individuals to achieve. Consequently, emerging interventions have expanded beyond direct pharmacological modulation of nicotinic receptors to target nicotine pharmacokinetics, addiction-related neural circuitry, and behavioral determinants of tobacco use. These approaches include nicotine-targeted immunotherapies, neuromodulation techniques, and digital health technologies designed to complement established cessation treatments and improve treatment accessibility, personalization, and long-term outcomes.

#### 4.6.1. Nicotine-Targeted Immunotherapies

Different immunotherapeutic strategies have been developed to alter the pharmacokinetics of nicotine before receptor engagement. These approaches aim to generate or administer nicotine-specific antibodies (NicAbs) that bind nicotine in the peripheral circulation, thereby reducing the amount and rate of nicotine entering the brain. By attenuating nicotine-induced activation of mesolimbic reward pathways without directly modulating nAChR function, immunotherapies represent a mechanistically distinct approach to the management of nicotine dependence [173,174]. Both active immunization using nicotine vaccines and passive immunization with nicotine-specific monoclonal antibodies (mAbs), such as Nic311, have been investigated, while combined active-passive immunization has also been explored as a strategy to enhance therapeutic efficacy [175,176]. Nicotine vaccines generally consist of a nicotine-derived hapten conjugated to an immunogenic carrier protein. Although clinical candidates such as NicVAX, NicQb, TA-NIC, and SEL-068 demonstrated promising early findings, their efficacy was not consistently reproduced in larger clinical trials, and no nicotine vaccine has received regulatory approval for smoking cessation [177]. A major limitation was the substantial interindividual variability in vaccine-induced antibody responses, with many participants failing to achieve antibody concentrations sufficient to meaningfully alter nicotine distribution [177]. Therapeutic efficacy may also depend on antibody affinity, specificity, binding capacity, and persistence rather than antibody titer alone. Furthermore, the rapid delivery of nicotine to the brain following cigarette inhalation and repeated nicotine exposure may exceed the binding capacity of circulating antibodies. Importantly, vaccines do not directly alleviate withdrawal symptoms, conditioned craving, or the behavioral and psychosocial components of tobacco dependence, which may further limit their effectiveness as stand-alone interventions [178].

Passive immunization with nicotine-specific mAbs may overcome some limitations of vaccination by providing immediate and more predictable antibody exposure with predefined affinity, specificity, and dose. However, high antibody concentrations may be required to neutralize repeated nicotine exposure, potentially necessitating repeated parenteral administration. Variability in antibody distribution and clearance, together with manufacturing costs and limited accessibility, may also restrict broad clinical implementation [179]. Combined active and passive immunization has therefore been investigated to integrate the sustained antibody production induced by vaccination with the immediate antibody availability provided by mAbs. In a preclinical study, combined treatment with a nicotine vaccine and Nic311 produced substantially greater attenuation of nicotine-induced locomotor sensitization (LMS) than either intervention alone, resulting in activity levels comparable to saline-treated controls [175]. This effect was achieved using a mean Nic311 dose of approximately 30 mg/kg, which was lower than the 80–160 mg/kg doses previously required to attenuate nicotine-related behaviors when the mAb was administered alone [176]. Nevertheless, total serum NicAb concentrations remained highly variable (58–274 μg/mL), reflecting differences in vaccine-induced antibody production and Nic311 pharmacokinetics [180]. Such variability may require individualized dosing or therapeutic monitoring to maintain effective antibody concentrations.

Overall, nicotine-targeted immunotherapies provide a biologically compelling strategy because they act upstream of nAChR activation and may avoid the receptor-mediated adverse effects associated with centrally acting pharmacotherapies. However, their clinical translation remains limited by heterogeneous vaccine responses, the rapid delivery and repeated exposure characteristics of inhaled nicotine, incomplete effects on withdrawal and conditioned smoking behaviors, and the high antibody concentrations required for effective nicotine sequestration. Future development may benefit from optimized hapten design, more immunogenic carrier and adjuvant systems, multivalent vaccines, and engineered mAbs with improved affinity, binding capacity, and circulating half-life. Nevertheless, future clinical studies will need to establish whether improved pharmacokinetic control of nicotine can produce clinically meaningful and sustained abstinence when immunotherapy is integrated with established pharmacological and behavioral interventions.

#### 4.6.2. Neuromodulation-Based Interventions

Neuromodulation techniques such as repetitive transcranial magnetic stimulation (rTMS) and transcranial direct current stimulation (tDCS) are non-invasive brain stimulation methods that have been investigated for tobacco cessation and are thought to modulate brain circuits involved in nicotine dependence [181]. rTMS uses magnetic pulses to stimulate cortical neurons and has been studied across 14 human studies, including 7 RCTs and 7 experimental studies [182,183,184,185]. Most studies (12/14) reported reductions in cue-induced craving, 7 reported decreased cigarette consumption, and 4/5 multi-session studies reported increased quit rates. In randomized trials, high-frequency rTMS (HF-rTMS) over the left dorsolateral prefrontal cortex (DLPFC) has been associated with reduced cigarette intake, reduced craving, and improved quitting outcomes [186]. Deep transcranial magnetic stimulation (deep TMS) has been reported to reduce smoking behaviour in clinical trials and has received FDA approval for tobacco cessation [185]. In contrast, tDCS has shown more inconsistent effects. Across 6 RCTs, 2 reported reduced craving, 1 reported reduced cigarette consumption, and none reported changes in quit rates, although some crossover studies reported reductions in craving and cigarette consumption [187,188]. Mechanistically, DLPFC-HF-rTMS has been associated with increased prefrontal activity and decreased activity in mesolimbic reward regions, including the medial orbitofrontal cortex and nucleus accumbens (NAc) [186]. Overall, rTMS is FDA-approved for tobacco cessation, whereas tDCS remains investigational, and further work is needed to optimize stimulation parameters and treatment approaches.

#### 4.6.3. Digital Health and Technology-Assisted Smoking Cessation

The persistent limitations of conventional tobacco cessation therapies have stimulated considerable interest in the development of digital technologies as complementary approaches for nicotine dependence management [189,190,191]. Advances in mobile health (mHealth), artificial intelligence (AI), virtual reality (VR), and augmented reality (AR) have created new opportunities to deliver personalized, scalable, and accessible interventions capable of supporting tobacco cessation efforts outside traditional healthcare settings. These technologies are particularly attractive for reaching younger populations and individuals with limited access to specialized tobacco cessation services. Representative digital health technologies, AI platforms, and immersive interventions investigated for tobacco cessation are summarized in Table 3. Smartphone-based tobacco cessation applications represent one of the most widely adopted forms of digital intervention. Applications such as, for example, Quit Genius [192], Smoke Free [193], and CureApp Smoking Cessation (CASC) combine behavioral counseling, progress tracking, craving management tools, and educational resources within user-friendly mobile platforms [191]. Clinical studies have demonstrated that these interventions can improve quit rates and treatment adherence, particularly when integrated with pharmacological therapies. Notably, the CASC platform combines a smartphone application with physician-support software and a portable carbon monoxide monitoring device, allowing users to visualize physiological improvements during smoking cessation and thereby enhancing motivation and treatment engagement [194].

AI has further expanded the capabilities of digital tobacco cessation programs through the development of adaptive and personalized behavioral interventions. AI-driven conversational agents and virtual coaches can provide real-time counseling, motivational interviewing, and relapse prevention strategies tailored to individual user characteristics. These systems may be particularly helpful during periods of heightened craving, withdrawal symptoms, and increased relapse risk, where timely behavioral intervention can influence treatment outcomes. For example, the QuitBot platform has demonstrated encouraging tobacco cessation outcomes by delivering automated, evidence-based behavioral support through natural language conversations [195]. In addition, emerging AI systems can estimate nicotine exposure, predict relapse risk, monitor behavioral patterns, and generate personalized educational content designed to increase awareness of smoking-related harms and reinforce cessation efforts. Such technologies have the potential to transform traditional tobacco cessation programs from static interventions into more dynamic and adaptive support systems.

Beyond AI-assisted behavioral interventions, immersive technologies such as VR and AR have emerged as innovative approaches for supporting tobacco cessation and relapse prevention. VR-based interventions allow individuals to experience simulated smoking-related environments while practicing coping strategies and craving management techniques in a controlled setting. Several pilot and proof-of-concept studies have demonstrated that repeated VR-based cue-exposure and behavioral training interventions can reduce smoking-related craving, improve self-efficacy, and strengthen coping responses to tobacco triggers, supporting the potential utility of immersive technologies in tobacco cessation programs [196,197,198]. VR systems have also been utilized to recreate high-risk situations, such as social gatherings or stress-inducing environments, enabling users to develop resistance to tobacco triggers before encountering them in real life [198]. Meanwhile, AR technologies offer the ability to overlay educational information and health-related visualizations onto real-world environments. Emerging AR applications can display personalized projections of smoking-related health consequences, estimate cumulative nicotine exposure, and visually demonstrate improvements associated with tobacco cessation, thereby enhancing risk perception and motivation to quit [199].

Although many of these technologies remain in early stages of clinical validation, accumulating evidence suggests that digital therapeutics, AI-driven behavioral support systems, and immersive VR/AR interventions may help overcome several limitations of current tobacco cessation therapies. Future large-scale RCTs will be required to establish their long-term efficacy, optimize integration with existing pharmacological treatments, and determine their role in precision tobacco cessation programs aimed at improving abstinence outcomes across diverse populations. Importantly, these technologies are not intended to replace established pharmacotherapies but may serve as complementary tools that enhance treatment adherence, personalize intervention strategies, and improve long-term abstinence outcomes when integrated with evidence-based tobacco cessation programs.

**Table 3:** Digital and technology-assisted interventions for smoking cessation and nicotine dependence. Emerging approaches, including smartphone-based applications, biomarker-integrated mobile platforms, AI conversational agents, machine learning-driven decision systems, and VR/AR interventions, are summarized together with their primary therapeutic functions and current evidence supporting their use as adjunctive tools for smoking cessation and relapse prevention.

Technology Category	Platform/Intervention	Primary Function	Current Evidence Level	Ref.
Smartphone Applications	Quit Genius	Digital cognitive behavioral therapy	RCTs	[192]
	Smoke Free	Behavioral support, craving management, quit tracking	RCTs and meta-analyses	[193]
	QuitSTART (National Cancer Institute)	Youth-oriented cessation support	Real-world implementation studies	[200]
	EX Program (Truth Initiative)	Personalized digital cessation coaching	Clinical and population studies	[201]
Smartphone Applications + Biomarker Monitoring	CureApp Smoking Cessation (CASC)	CO monitoring, physician support, behavioral intervention	RCTs	[194]
	Pivot Program	Breath sensor and app-based coaching	Prospective clinical studies	[202]
	Bupa Quit Coach	Digital coaching with progress tracking	Observational studies	[202]
AI Conversational Agents	QuitBot	AI-driven counseling and motivational support	Early clinical trials	[195]
	Bella (WHO Digital Health Initiative)	Conversational smoking cessation support	Pilot implementation studies	[203]
	ChatGPT-based cessation assistants	Personalized educational and behavioral support	Emerging feasibility studies	[204]
Machine Learning Systems	Relapse Prediction Models	Predict relapse risk and personalize treatment	Early-stage clinical validation	[205]
	Just-In-Time Adaptive Interventions	Deliver support during high-risk moments	Pilot and feasibility studies	[206]
	Predictive Behavioral Analytics Platform (1D-CNN model)	Treatment optimization and adherence monitoring	Emerging clinical research	[207]
Virtual Reality (VR)	VR Cue-Exposure Therapy	Craving reduction and trigger	Pilot studies and RCTs	[198]
	VR Behavioral Skills Training	Coping skills practice in simulated environments	Pilot studies	[198]
	VR-Based Relapse Prevention Programs	Exposure to high-risk smoking situations	Feasibility and clinical studies	[208]
	Virtual Therapeutic Community Platforms	Behavioral modification and addiction recovery	Early-stage evaluation	[209]
Augmented Reality (AR)	AR Health Visualization Platforms	Visualization of smoking-related health consequences	Emerging behavioral studies	[210]
	AR Smoking Trigger Recognition Systems	Real-time identification of smoking cues	Pilot studies	[211]
	AR Educational Smoking Prevention Tools	Youth-focused nicotine education	Feasibility studies	[212]

### 4.7. Current Guideline Recommendations

Recent guidelines from major organizations recommend an evidence-based approach integrating behavioural support with pharmacological treatment for tobacco cessation. The 2024 World Health Organization (WHO) Clinical Treatment Guideline for Tobacco Cessation in Adults recommends combining pharmacotherapy and behavioural interventions to support tobacco users interested in quitting [213]. WHO recommends that healthcare providers routinely provide brief advice to tobacco users accessing healthcare settings, while individuals interested in quitting should be offered more intensive behavioural support, including individual face-to-face counselling, group counselling, or telephone counselling.

For pharmacological management, WHO strongly recommends varenicline, NRT, bupropion, and cytisine as treatment options for tobacco users who smoke and are interested in quitting. Varenicline, NRT, and bupropion are recommended as first-line options, while cytisine is also recognized as a recommended pharmacological option. WHO further supports combination treatment strategies, including combination NRT consisting of a nicotine patch with a short-acting formulation such as gum or lozenges, and the use of bupropion in combination with NRT or varenicline when there is an inadequate response to first-line treatments.

The 2025 Canadian guideline from the Canadian Task Force on Preventive Health Care, published in the Canadian Medical Association Journal (CMAJ), recommends that all adults who smoke tobacco cigarettes should be encouraged to quit and offered one or more evidence-based cessation interventions [214]. The guideline identifies behavioral interventions, pharmacotherapy, and combined approaches as recommended options and emphasizes shared decision-making to help individuals select cessation strategies that align with their values and preferences.

These recommendations are consistent with other major international guidelines. The 2025 National Institute for Health and Care Excellence (NICE) guideline recommends providing behavioral support and pharmacological treatments, including nicotine replacement therapy, varenicline, bupropion, and cytisine, for individuals who wish to stop smoking [215]. Similarly, the 2021 United States Preventive Services Task Force (USPSTF) recommends that clinicians ask adults about tobacco use, advise cessation, and provide behavioral interventions and FDA-approved pharmacotherapy for nonpregnant adults who use tobacco [216]. Regarding ENDSs, NICE recommends that adults interested in using nicotine-containing e-cigarettes for smoking cessation receive clear and consistent information regarding their use, including uncertainties related to long-term health effects, while USPSTF considers the evidence insufficient to determine the balance of benefits and harms of e-cigarettes for tobacco cessation.

## 5. Limitations and Translational Challenges of Current and Emerging Therapeutic Strategies

Despite remarkable progress that has been made in the development of interventions for the treatment of smoking addiction, sustaining abstinence remains a challenging feat. Although pharmacological medications, including NRTs, varenicline, cytisine/cytisinicline, and bupropion, increase the likelihood of success relative to quitting without any help, these interventions tend to underperform in actuality. The efficiency of these therapies depends on such parameters as medication adherence, level of nicotine dependence, presence of concurrent psychological disorders, access to psychological help, socio-economic background, and the patients’ desire to quit smoking [217,218]. Furthermore, relapse is common especially during the first year following abstinence from tobacco use, suggesting that reducing withdrawal and overcoming acute cravings does not guarantee sustained protection against future nicotine usage. Every treatment strategy has clinical and practical limitations [219].

For example, NRT is effective in providing nicotine intake while protecting from other harmful combustion products; however, it might be hindered by inadequate dosage, lack of adherence, and slower nicotine delivery than regular cigarette smoking. While a combination of NRTs could provide more effective management of withdrawal and breakthrough cravings, it would not help in tackling conditioned stimuli of smoking and behavioral habits associated with tobacco use. Varenicline and cytisine/cytisinicline alleviate withdrawal symptoms and decrease nicotine reinforcement through partial agonism of α4β2-containing nicotinic acetylcholine receptors; however, it could be ineffective for some people and side effects like nausea, sleep disruption, and unusual dreams could pose obstacles to treatment adherence. Bupropion gives an alternative treatment through dopaminergic and noradrenergic neurotransmission alteration and nicotinic receptor antagonism, yet it is less efficient and has certain contraindications. Therefore, treatment decision-making should be based on multiple parameters of efficacy, tolerability, safety, accessibility, and preferences rather than a single cure-all therapy [220].

The limitations of current pharmacotherapies also reflect the biological complexity of nicotine addiction. Although α4β2-containing nAChRs have a central role in nicotine reinforcement and remain an important therapeutic target, nicotine dependence is not mediated by a single receptor subtype or neural pathway. Other nAChR subtypes and neural circuits involved in reward, aversion, stress, learning, cognitive control, and withdrawal contribute to continued nicotine use and relapse. Behavioral routines, environmental cues, emotional states, and social factors may also persist after acute withdrawal has subsided. Consequently, greater receptor subtype selectivity may improve pharmacological precision but may not necessarily produce superior long-term cessation outcomes. Next generation of orthosteric ligands and allosteric modulators should therefore be evaluated not only by receptor affinity, selectivity, and preclinical activity but also by their ability to improve sustained abstinence, relapse prevention, safety, and tolerability [133].

Emerging therapies offer opportunities to target mechanisms not adequately addressed by conventional pharmacotherapies but also face important translational barriers. Nicotine vaccines and nicotine-specific mAbs aim to sequester nicotine in the peripheral circulation and reduce its access to the brain. However, previous vaccine studies (for example, Carson et al. [221] and references therein) demonstrated substantial variability in antibody concentration, affinity, binding capacity, and persistence. In addition, the rapid delivery and repeated exposure associated with cigarette smoking may exceed the nicotine-binding capacity of circulating antibodies. The mAbs may provide more predictable exposure, but high dose requirements, repeated administration, manufacturing complexity, and cost could limit widespread use. Importantly, immunotherapies do not directly address nicotine withdrawal, craving, and behavioral aspects of tobacco addiction; therefore, they can be more suitable as adjunctive rather than individual therapies [221].

Neuromodulation techniques have demonstrated potential in diminishing cravings and influencing neural circuits associated with reward and cognitive control. However, variations in stimulation targets, treatment protocols, participant characteristics, and outcome measures have led to inconsistent results. The longevity of treatment effects is still unclear, and the requirement for specialized equipment, trained staff, and multiple sessions may restrict accessibility. To determine the clinical significance and practicality of these methods, standardized protocols and extended clinical studies are necessary [222].

Recent developments in digital technology offer promising alternatives to help people quit smoking, but many applications lack sufficient clinical validation. Most studies suffer from small sample sizes, inadequate follow-up duration, high attrition rates, low long-term engagement, and reliance upon self-reported rather than biochemically confirmed outcomes. Further issues include concerns regarding data privacy, algorithmic transparency, digital literacy, disparities in access, and regulatory oversight. Finally, while short-term engagement or reduced smoking may appear beneficial, this does not equate to sustained therapeutic efficacy [223].

Certain subpopulations remain underrepresented in smoking cessation research, including adolescents, pregnant individuals, those with psychiatric or substance use disorders, highly nicotine-dependent users, and socioeconomically disadvantaged groups. In addition, the proliferation of diverse nicotine delivery devices adds another layer of complexity, since dependency profiles can vary widely depending upon whether one uses combustible cigarettes, ENDSs, nicotine pouches, or several different types of products. Therefore, evidence drawn largely from conventional cigarette smokers likely fails to capture the full scope of modern-day nicotine usage or prevailing treatment demands [224].

Overall, major breakthroughs in addressing nicotine dependence management likely require individualised approaches integrating pharmacotherapies with behavioural intervention and incorporating novel interventions where there is strong evidence of their efficacy. Future research should focus more strongly on measures such as sustained biochemical verification of tobacco-free status, relapse prevention, long-term safety, adherence, availability and affordability. Direct comparison of different therapeutic modalities and combinations would help determine if novel therapies provide added benefit over existing ones.

## 6. Conclusions

Although currently available pharmacotherapies have led to meaningful improvements in tobacco cessation outcomes, nicotine addiction continues to be recognized as a chronic and relapsing disorder, with a substantial proportion of individuals experiencing relapse despite treatment. This limited long-term efficacy reflects the multifactorial nature of nicotine dependence, which arises from complex interactions among receptor pharmacology, neural circuitry, genetic susceptibility, psychological factors, and social determinants of health. While the pharmacological targeting of nicotinic acetylcholine receptors has provided the foundation for current tobacco cessation therapies, existing approaches remain insufficient to fully address the heterogeneity of nicotine addiction observed across diverse populations.

Recent advances in structural biology, neuropharmacology, and receptor signaling have created new opportunities for the development of more selective and potentially more effective therapeutic agents. In particular, growing interest has been directed toward subtype-selective nAChR ligands, positive and negative allosteric modulators, immunotherapeutic approaches such as nicotine vaccines and monoclonal antibodies, and novel neuromodulatory interventions. Nevertheless, the translation of promising preclinical findings into clinically successful therapies has remained challenging, with many investigational compounds failing to demonstrate meaningful advantages over existing treatments in large-scale clinical trials. This gap underscores the need for more robust translational frameworks that better integrate mechanistic insights with clinically relevant endpoints.

Furthermore, increasing recognition of interindividual differences in treatment response suggests that a universal approach to tobacco cessation is unlikely to achieve optimal outcomes. Future therapeutic strategies will likely benefit from personalized medicine approaches that incorporate genetic, neurobiological, behavioral, and environmental factors to guide treatment selection. Simultaneously, the integration of pharmacological interventions with behavioral therapies, digital health technologies, and public health initiatives may further enhance treatment adherence and long-term abstinence. Emerging AI-assisted mobile applications, digital therapeutics, and immersive VR/AT platforms may further support personalized tobacco cessation strategies by providing adaptive behavioral support, relapse prevention tools, and improved patient engagement. From a global perspective, future research and therapeutic development must also address persistent disparities in access to tobacco cessation resources, particularly in low- and middle-income countries, where the burden of tobacco use remains substantial and access to evidence-based interventions is often limited. Ensuring equitable access to effective cessation therapies, while adapting interventions to diverse cultural and socioeconomic contexts, will be essential for reducing global tobacco-related morbidity and mortality.

Overall, while current pharmacotherapies have established a valuable foundation for the treatment of nicotine dependence, there remains a pressing need for continued innovation in the field. Advances in medicinal chemistry, receptor pharmacology, neurobiology, and precision medicine are expected to drive the development of safer, more effective, and more targeted therapeutic options. Continued investment in multidisciplinary research and large-scale clinical trials will be critical for translating these advances into meaningful clinical outcomes and ultimately reducing the worldwide burden of nicotine addiction and tobacco-related morbidity and mortality, with a reduction in burden on health systems, the environment and national economies.

## Figures and Tables

**Table 2:** Representative nicotinic receptor ligands and antidepressant agents investigated for tobacco cessation therapy. The table shows the chemical structures and reports binding affinities or inhibitory activities toward the α4β2 nicotinic acetylcholine receptor (nAChR) subtype as receptor binding affinities (*K_i_*) or inhibitory potencies (IC_50_).

Agent	Structure	α4β2 Binding/Inhibition Value *	Ref
Cytisine	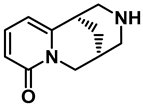	*K_i_* = 0.23 nM	[147]
Varenicline	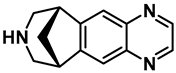	*K_i_* = 0.15 nM	[147]
Dianicline	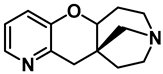	*K_i_* = 10 nM	[127]
ABT-089	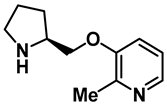	*K_i_* = 76 nM	[148]
TC-2559	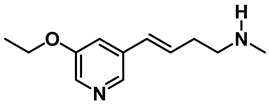	*K_i_* = 5 nM	[133]
Bupropion	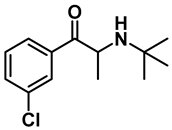	IC_50_ = 8 µM	[149]
Nortriptyline	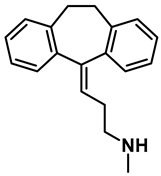	IC_50_ = 100 nM	[150]

* Values are reported as described in the original studies and may reflect different experimental methodologies.

## Data Availability

No new data were created or analyzed in this study. Data sharing is not applicable to this article.
